# Complete Genome Sequence of Bacillus licheniformis TAB7, a Compost-Deodorizing Strain with Potential for Plant Growth Promotion

**DOI:** 10.1128/MRA.01659-18

**Published:** 2019-01-24

**Authors:** Enock Mpofu, Felipe Vejarano, Chiho Suzuki-Minakuchi, Yoshiyuki Ohtsubo, Masataka Tsuda, Joydeep Chakraborty, Masatoshi Nakajima, Kazunori Okada, Nobuki Tada, Toshiaki Kimura, Hideaki Nojiri

**Affiliations:** a Biotechnology Research Center, The University of Tokyo, Tokyo, Japan; b Collaborative Research Institute for Innovative Microbiology, The University of Tokyo, Tokyo, Japan; c Graduate School of Life Sciences, Tohoku University, Sendai, Japan; d Department of Applied Biological Chemistry, The University of Tokyo, Tokyo, Japan; e Toyota Motor Corporation, Nagoya, Japan; University of Delaware

## Abstract

Bacillus licheniformis strain TAB7 degrades short-chain fatty acids responsible for offensive odor in manure and is used as a deodorant in a compost-deodorizing technology. Here, we report the complete genome sequence of strain TAB7, which consists of a 4.37-Mb chromosome and two plasmids (42 kb and 31 kb).

## ANNOUNCEMENT

**FIG 1:**
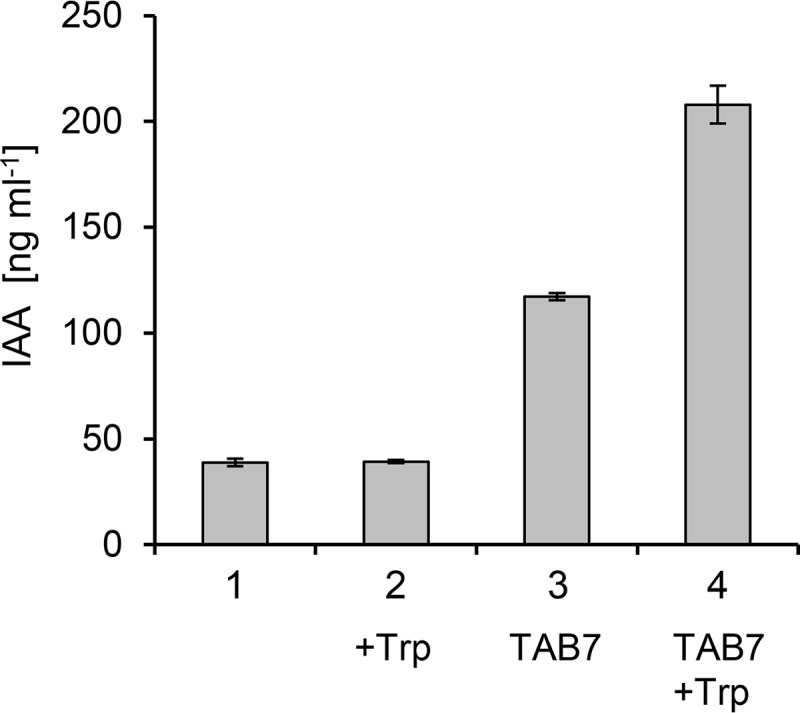
IAA production by Bacillus licheniformis TAB7. TAB7 cells were grown in LB at 30°C for 24 h with shaking (300 strokes/min) and then centrifuged. The supernatant was then acidified, and IAA was extracted by acetonitrile. Liquid chromatography-tandem mass spectrometry (LC-MS/MS) analysis was performed on a Xevo TQ (Waters) platform using an Acquity ultraperformance liquid chromatography (UPLC) ethylene bridged hybrid (BEH) C_18_ column (1.7 µm, 2.1 by 50 mm; Waters). A 10-ng aliquot of ^13^C-labeled IAA was used as an internal standard. 1, sterile LB (control); 2, sterile LB with 300 μg/ml of tryptophan (Trp, control); 3, extracts from the TAB7 culture; 4, extracts from the TAB7 culture supplemented with 300 μg/ml of tryptophan. Data are expressed as means ± standard deviation of results from technical triplicates.

TAB7 is a thermophilic Bacillus licheniformis strain isolated as a Tween 20 (a surfactant with a fatty acid side chain) degrader from composting manure in Japan (1). It degrades short-chain fatty acids responsible for the offensive odor in compost and is commercially available as a deodorizing agent for composts (2). Several Bacillus spp. can produce indole-3-acetic acid (IAA) and promote plant growth (3–5). Our cultivations of TAB7 in lysogeny broth (LB) (6), with and without tryptophan, resulted in production of IAA in both cases (Fig. 1). Thus, TAB7 may not only deodorize compost but may also promote plant growth. Therefore, its genome sequence will be useful in comparative genomic studies with known plant growth-promoting bacteria.

For genome sequencing, TAB7 cells were grown overnight in 10 ml of LB at 30°C with shaking (300 strokes/min). DNA was extracted and purified using a Wizard genomic DNA purification kit (Promega) following the manufacturer’s instructions. Sequencing with a v3 chemistry 600-cycle kit (Illumina) was done using a MiSeq sequencer with PCR-free paired-end (PE) and mate pair (MP) libraries that were prepared with TruSeq DNA PCR-free and Nextera mate pair library preparation kits (Illumina), respectively, following the manufacturer’s instructions. Obtained reads were processed with ShortReadManager 0.995 (7) to extract paired reads, perform low-abundance 21-mer-read trimming, and discard reads shorter than 150 and 100 bp in the PE and MP data sets, respectively. One million PE (273 Mb) and 0.7 million MP (128 Mb) reads were assembled with Newbler 2.8 (Roche) into three scaffolds, two of which consisted of single circular contigs without gaps (plasmids). A total of 29 repeat-induced gaps in the remaining scaffold (chromosome) were identified using GenoFinisher 2.1 (7), and their precise sequences and locations with respect to other contigs were determined with AceFileViewer 1.5 (7), using the MP data. Assembled replicons were checked for errors with the GenoFinisher tool FinishChecker (8), confirming complete gap resolution.

A 4,367,367-bp chromosome (85× coverage) and two circular plasmids, pTAB7A and pTAB7B (42,138 bp and 31,204 bp, with 290× and 400× coverage, respectively) were assembled. Open reading frame (ORF) prediction and annotation were done using the Microbial Genome Annotation Pipeline (MiGAP) (9) and the NCBI Prokaryotic Gene Annotation Pipeline (PGAP) (10). The two annotation results were compared and manually corrected using GenomeMatcher (11) and then merged. The Kyoto Encyclopedia of Genes and Genomes (KEGG) pathway database (12) was used to predict metabolic pathways.

The chromosome of TAB7 has a G+C content of 45.82% and bears 4,429 coding DNA sequences (CDS). It contains 85 tRNA genes and 7 rRNA operons. pTAB7A (51 CDS) and pTAB7B (33 CDS) have G+C contents of 40.10% and 38.61%, respectively. The TAB7 chromosome harbors nitrilase (*yhcX*) and IAA-acetyl-transferase (*ysnE*) (4) genes that may be involved in IAA biosynthesis. It also has genes encoding phenolic acid decarboxylase (*padC*) (13), vanillic acid decarboxylase (*vdcC*) (14), and protocatechuic acid-degrading enzymes (*praABCDEHI*) (15), which are involved in catabolism of phenolic compounds known to have negative allelopathy on some plants (16–18). Furthermore, putative genes involved in the production of other plant growth-promoting compounds, such as bacillibactin (*dhbABCEF*), and acetoin (*alsDRS*) (4, 19) were found, further suggesting TAB7 involvement in plant growth promotion.

### Data availability.

The genome sequence of Bacillus licheniformis strain TAB7 has been deposited in DDBJ/ENA/GenBank under the accession numbers CP027789 (chromosome), CP027790 (pTAB7A), and CP027791 (pTAB7B). Raw sequencing data have been deposited under BioProject accession number PRJNA438467. Details of the assembly procedure for the generation of the complete sequences and the parameters used with each software are available in the comment section of each submission as part of the metadata.
